# Generation of functionally active resident macrophages from adipose tissue by 3D cultures

**DOI:** 10.3389/fimmu.2024.1356397

**Published:** 2024-06-21

**Authors:** Adèle Arlat, Marie-Laure Renoud, Jean Nakhle, Miguel Thomas, Jessica Fontaine, Emmanuelle Arnaud, Cédric Dray, Hélène Authier, Paul Monsarrat, Agnès Coste, Louis Casteilla, Marielle Ousset, Béatrice Cousin

**Affiliations:** ^1^ RESTORE Research Center, Université de Toulouse, INSERM 1301, CNRS 5070, Etablissement Français du Sang (EFS), Ecole Nationale Vétérinaire de Touloue (ENVT), Toulouse, France; ^2^ Dental Faculty and Hospital of Toulouse – Toulouse Institute of Oral Medicine and Science, CHU de Toulouse, Toulouse, France; ^3^ Artificial and Natural Intelligence Toulouse Institute (ANITI), Toulouse, France

**Keywords:** resident macrophage, macrophage subpopulation, adipose tissue, bone marrow, 3D culture, metabolism, phagocytosis, unsupervised analysis

## Abstract

**Introduction:**

Within adipose tissue (AT), different macrophage subsets have been described, which played pivotal and specific roles in upholding tissue homeostasis under both physiological and pathological conditions. Nonetheless, studying resident macrophages *in-vitro* poses challenges, as the isolation process and the culture for extended periods can alter their inherent properties.

**Methods:**

Stroma-vascular cells isolated from murine subcutaneous AT were seeded on ultra-low adherent plates in the presence of macrophage colony-stimulating factor. After 4 days of culture, the cells spontaneously aggregate to form spheroids. A week later, macrophages begin to spread out of the spheroid and adhere to the culture plate.

**Results:**

This innovative three-dimensional (3D) culture method enables the generation of functional mature macrophages that present distinct genic and phenotypic characteristics compared to bone marrow–derived macrophages. They also show specific metabolic activity and polarization in response to stimulation, but similar phagocytic capacity. Additionally, based on single-cell analysis, AT-macrophages generated in 3D culture mirror the phenotypic and functional traits of *in-vivo* AT resident macrophages.

**Discussion:**

Our study describes a 3D *in-vitro* system for generating and culturing functional AT-resident macrophages, without the need for cell sorting. This system thus stands as a valuable resource for exploring the differentiation and function of AT-macrophages *in vitro* in diverse physiological and pathological contexts.

## 1 Introduction

Adipose tissue (AT) is not only a passive storage depot for energy excess but also a vital endocrine and immune organ (1). Indeed, AT communicates with all other organs through an active release of various adipokines and inflammatory factors that significantly influence overall energy balance in the body and metabolic health (2). In addition to mature adipocytes, AT harbors a diverse and plentiful immune cell population, extensively investigated, with macrophages emerging as the predominant cell type (3). Macrophages play a critical role in triggering immune responses by detecting initial insults and releasing an array of inflammatory mediators, including cytokines and chemokines (4). Their biology is intricate and influenced by various factors such as tissue environment, ontogeny, and their inflammatory status within their respective niches (5, 6). Like in most tissues, AT-macrophages form a diverse population, with resident AT-macrophages believed to primarily originate from embryonic yolk-sac precursors under normal conditions, exhibiting the ability to self-renew through proliferation (7, 8). Another subset of AT-macrophages arises after the final differentiation of monocytes [bone marrow (BM)–derived macrophages] (9). In most tissues, resident and BM-derived macrophage subsets can be distinguished using four markers, namely, *Timd4*, *Folr2*, and *Lyve1* for identifying resident macrophages, while *Ccr2* is proposed for identifying BM-derived macrophages (10). A recent analysis of macrophage heterogeneity identified CD206 as a marker of resident macrophages in AT (11) and dermis (12).

Depending on their origin and their environment, macrophages display specific functional characteristics (13). In the physiological state, resident macrophages play a critical role in maintaining tissue homeostasis (14). In the skin, resident dermal macrophages have been described to coordinate defense against infection, to play critical roles in all stages of healing process, or to contribute to stress-induced hair follicle regeneration (15–17). In the AT, macrophages play a critical role in a variety of physiological and pathological processes. In a physiological state, resident AT-macrophages are considered metabolically advantageous. They play crucial roles in maintaining AT homeostasis, tissue expansion, and remodeling (18), as well as tissue regeneration (19). Conversely, BM-derived macrophages can negatively impact AT function by promoting inflammation, insulin resistance, and fibrosis (20, 21). The distinct roles of various AT-macrophages subsets highlight the importance of their imprinting by the cellular niche. This prompts our investigation into their intrinsic properties.

Unfortunately, studying resident macrophages *in vitro* presents a challenge due to the lack of an adequate culture system that can produce enough cells that accurately reflect *in-vivo* characteristics. Furthermore, sorting resident macrophages usually alters their intrinsic properties. Additionally, the preservation of their properties in culture for an extended period while maintaining their characteristics is a challenging task due to the tissue microenvironment’s critical role in macrophage imprinting (22). Therefore, an *in-vitro* approach that supports the production of resident macrophages seems essential.

In this study, we adapted an efficient method initially developed for the production of mature and functional AT mastocytes (23) to generate mouse AT resident macrophages. Our research demonstrates that cultivating AT stroma-vascular cells in a three-dimensional (3D) environment generates fully developed and functional AT-macrophages with distinct transcriptomic, phenotypic, metabolic, and functional characteristics compared to BM-derived macrophages *in vitro*. These cultured cells successfully recapitulate the characteristics observed *in vivo* for resident AT-macrophages.

## 2 Material and methods

### 2.1 Animals

Experiments were performed on 6- to 8-week-old male C57Bl6/J Hola HSD mice (ENVIGO, Gannat, France). Animals were group-housed in a controlled environment (12h light/dark cycles at 21°C) with unrestricted access to water and a standard chow diet in a pathogen-free animal facility. Mice were killed by cervical dislocation and AT and femur were removed.

The animal study was approved by European Community Guidelines (2010/63/UE)/institutional ethics committee N.122 US006/CREFRE. The study was conducted in accordance with the local legislation and institutional requirements.

### 2.2 Isolation of adipose derived stromal vascular cells fraction and bone marrow cells

Sub-cutaneous adipose tissues (sc-AT) were collected, and lymph nodes removed. Femurs were collected and immediately flushed with α-MEM medium (Gibco, Thermo Fisher Scientific, Waltham, USA). Sc-AT sustained a mechanical dissociation and enzymatic digestion at 37°C with collagenase (NB4, Sigma-Aldrich, Saint-Louis, USA) for 30 min. After filtration and centrifugation, stroma vascular cells were isolated as described previously (19). A red blood cells lysis (NH4Cl 155 mM, K2HPO4 5.7 mM, EDTA 0.1 mM) was performed on BM and adipose stromal vascular fraction (SVF), and cells were then counted with cell counter (Beckman Coulter, Brea, USA).

### 2.3 *In vitro* production of adipose tissue macrophages

Adipose SVF cells were seeded on ultra-low adherence 96 wells round bottom plates (COSTAR, Dutcher, Bernolsheim, France) (10^5^ cells/well) with Roswell Park Memorial Institute (RPMI, InVitrogen, Thermo Fisher Scientific, Waltham, USA) supplemented with Glutamax medium (Gibco, Thermo Fisher Scientific, Waltham, USA), Heat Inactivated Newborn Calf Serum (hiNBSC; Gibco) (10%), a cocktail of amphotericin (Invitrogen, Thermo Fisher Scientific, Waltham, USA), streptomycin, and penicillin (1%; Life Technologies, Carlsbad, USA) and macrophage colony-stimulating factor (M-CSF) (10 ng/ml; PeproTech, Thermo Fisher Scientific, Waltham, USA). Cells were briefly centrifuged and incubated at 37°C with 5% CO_2_. After 4 days of culture, the cells spontaneously aggregate to form spheroids. Starting on day 7, the cells begin to spread out of the spheroid and adhere to the culture plate.

### 2.4 *In vitro* macrophages culture

BM cells were seeded on non-adherent Petri dishes at a concentration of 2.10^6^ cells in RPMI supplemented with Glutamax medium (Gibco, Thermo Fisher Scientific, Waltham, USA), hiNBSC (Gibco, Thermo Fisher Scientific, Waltham, USA) (10%), a cocktail of amphotericin (Invitrogen, Thermo Fisher Scientific, Waltham, USA), streptomycin, and penicillin (Thermo Fisher Scientific, Waltham, USA) (1%) and M-CSF (Pepro Tech, Thermo Fisher Scientific, Waltham, USA) (10 ng/ml). Cells were then incubated at 37°C with 5% CO2.

After 13 days in culture, differentiated BM-macrophages and cells harvested from AT-3D cultures were counted and seeded on adherent culture plates with RPMI supplemented with Glutamax medium (Gibco, Thermo Fisher Scientific, Waltham, USA), hiNBSC (Gibco, Thermo Fisher Scientific, Waltham, USA) (10%) and a cocktail of amphotericin, streptomycin, and penicillin (1%) overnight. Macrophages were then treated or not with interleukine-4 (IL-4; PeproTech, Thermo Fisher, Waltham, USA) (10 ng/ml) or interferon-γ (IFN-γ; PeproTech, Thermo Fisher, Waltham, USA) (50 ng/ml) for 4h (gene expression) or 24h (protein expression), or used for flow cytometry analyses or functional assays [phagocytosis and reactive oxygen species (ROS) production].

### 2.5 Spheroid dissociation

After 7, 13, or 21 days in culture, spheroids were collected and dissociated with NB4 (1.7 U/ml; Sigma-Aldrich, Saint-Louis, USA) and dispase I (2.5 U/ml; Sigma-Aldrich, Saint-Louis, USA) at 37°C for 40 min. Spheroid were then mechanically dissociated with a pipette and cells were centrifuged to complete dissociation.

### 2.6 Immunohistochemistry and microscopy

After 13 days, cells that had spread around the spheroids were seeded onto adherent culture plates, fixed with paraformaldehyde (PFA; Sigma-Aldrich, Saint-Louis, USA) (2%) and permeabilized with saponin (0.25%) (Sigma-Aldrich). Unspecific binding sites were blocked using fetal bovin serum (Sigma-Aldrich, Saint-Louis, USA) before incubation overnight at 4°C with primary antibody rat anti-mouse F4/80 (Bio-Rad, Hercules, USA). Cells were then washed and stained with a secondary Alexa Fluor 488–coupled donkey anti-rat antibody (Invitrogen, Thermo Fisher Waltham, USA), and 4’,6-diamidino-2-phenylindole (DAPI) (Sigma-Aldrich, Saint-Louis, USA). Images were obtained with Opera Phenix (PerkinElmer, Waltham, USA) and analyzed with Harmony software.

### 2.7 Flow cytometry analysis and cell sorting

AT- and BM-macrophages, dissociated spheroid cells, and sc-AT SVF cells were incubated with live/dead reagent (Thermo Fisher Scientific, Waltham, USA) and FcR-blocking reagent (BD Pharmigen, Franklin Lakes, USA) (except for CD16/32 staining). Cells were then stained with conjugated rat anti-mouse antibodies and the specificity of each staining was checked using specific isotype controls (**Supplementary Table S1**). After extracellular staining, cells were washed with MACS buffer (Miltenyi Biotec, Bergisch Gladbach, Germany). For intracellular staining, cells were fixed with 2% PFA (Sigma-Aldrich, Saint Louis, USA) solution and permeabilized with 0.5% Saponin (Sigma Aldrich, Saint Louis, USA) before incubating with coupled intracellular antibodies diluted in 0.5% Saponin for 40 min. Events were acquired on flow cytometer (LSRFortessa, BD Biosciences, Franklin Lakes, USA), and data were analyzed using Kalusa version 1.2 (Beckman Coulter, Brea, USA).

For unsupervised analyses, preprocessing of cytometry data was realized using FlowJo 10.8.1 software (Flo Jo LLC, Becton Dickinson, Franklin Lakes, USA). Arc sinh normalization of concatenated individual flow cytometry standard files and homogenization by downsampling of each sample were performed before gating on alive, CD45^+^. Then we selected F4/80^+^, CD11b^+^ cells. A nonlinear dimensionality reduction [Uniform Manifold Approximation and Projection (UMAP)] and Louvain clustering on Python was performed to segregate populations based on the expression of specific macrophage markers (CD45, F4/80, CD11b, CD206, CD16/32, MHC-II, MerTK, Dectin-1, and CD36).

For macrophage sorting experiments, sc-AT SVF cells were stained with DAPI, CD45, CD11b, and F4/80 antibodies (**Supplementary Table S1**). DAPI^−^/CD45^+^/CD11b^+^/F4/80^+^ cells were sorted (BD FACS ARIA Fusion III Cell sorter, BD Biosciences, Franklin Lakes, USA) and directly incubated in lysis buffer (Promega, Madison, USA) for subsequent analysis.

### 2.8 RNA extraction, real-time qPCR, and bulk RNA-seq

RNA was extracted from sorted sc-AT macrophages, AT- and BM-cultured macrophages, and purified using a Promega extraction kit (Relia PreP™ RNA Cell Miniprep, Promega, Madison, USA). For real-time PCR, 250 ng of total RNA were reverse transcribed using the High-Capacity cDNA Reverse Transcription kit (Applied Biosystems, Thermo Fisher Scientific, Waltham, USA). cDNAs were diluted and mixed with FAST SYBR GREEN (Roche Diagnostics, Meylan, France), and primers (**Supplementary Table S2**) on a LightCycler 480 (Roche Applied Science, Penzberg, Germany). All relative gene expression was determined using a standard curve realized with cDNA dilutions (1/5, 1/10, 1/20, and 1/40) and the ratio with GAPDH reporter gene.

For bulk RNA-seq, RNA quality was assessed using HS RNA Kit on Fragment analyser (Agilent Technologies, Santa Clara, USA). Analysis was performed in triplicate/duplicate of total RNAs from AT- and BM-cultured macrophages and sc-AT–sorted macrophages. cDNA libraries were generated according to Illumina Stranded mRNA Prep kit (Illumina, San Diego, USA) from 100 ng tRNA. cDNA was amplified for 13 cycles and barcoded with IDT^®^ for Illumina^®^ RNA UD Indexes Set A, Ligation (96 indexes, 96 samples). Libraries were tested for quality using HS NGS Fragment kit on Fragment Analyzer (Agilent Technologies, Santa Clara, USA) and concentrations were measured using KAPA qPCR library quantification kit (Roche Diagnostics, Meylan, France). Samples were pooled together to 8 nM concentration and sequenced with an Illumina NovaSeq SP 200 using 2 × 100 bp mode. RNA-seq reads were processed using the Galaxy web-based platform (https://usegalaxy.eu/). RNA-seq data were obtained in FASTQ format, reads were trimmed using Trim Galore! (24), and read quality was analyzed by FASTQC (25). Trimmed reads were mapped to the Mus musculus genome assembly GRCm39 using HISAT2 (26). Reads were assigned to genes in the custom GTF files and counted using featureConts (27).

Analysis was performed through R/RStudio. Hierarchical gene clustering was achieved using *multiClust* package. Differential analysis of gene expression between AT and BM was performed using *DESeq2* package with False Discovery Rate at the adjusted *p*-value level of 0.001 to counteract gene expression linked to a culture effect. Using only the previous significant genes, principal components analysis (PCA) was performed on normalized counts for all samples. To functionally group genes weighting for each PCA dimension, Gene Set Enrichment Analysis (GSEA) on biological processes was run using *clusterProfiler* package and the respective contribution of each gene in each PCA dimension as the ranking criteria. Over representation analysis (ORA) was performed through the *goseq* package. Bulk RNA-seq datasets were deposited in the ArrayExpress repository under the following accession numbers E-MTAB-14120.

### 2.9 Cytokine and NO production analysis

After 24h of treatment (cf. *in-vitro* macrophages culture) supernatants of AT- or BM-macrophage cultures were collected and cytokines, chemokines, or NO production was measured. Cytokine and chemokines production was analyzed with Mouse Macrophage/Microglia LEGENDPlex™ fluorescence bead-based immunoassay (BioLegend, San Diego, USA) to probe and quantify the levels of seven soluble factors (CXCL1, TGF-β1, CCL22, IL-12p70, IL-6, TNF-α, and IL-12p40) following supplier’s protocol. Raw data were given as the MFI of phycoerythrin (PE) signal. The concentration (pg/ml) of each soluble factor present in culture supernatants was determined by comparing the PE MFI for each target against seven individual protein standards. Data were analyzed using LEGENDplex™ v.8.1 data analysis software (BioLegend, San Diego, USA). NO production was quantified using Griess reagent. Absorbances were measured in ENVISION (PerkinElmer, Waltham, USA) at 630 nm.

### 2.10 Metabolic flux analysis

AT- and BM-macrophages were seeded at 20,000 cells/well in a poly-D-lysine–coated Seahorse XFe96 cell culture microplate (Agilent Technologies, Santa Clara, USA) in complete RPMI/Glutamax medium (Gibco, Thermo Fisher Scientific, Waltham, USA) supplemented with Heat Inactivated Newborn Calf Serum (hiNBSC; Gibco, Thermo Fisher Scientific, Waltham, USA) (10%) and a cocktail of amphotericin (Invitrogen, Thermo Fisher Scientific, Waltham, USA), streptomycin, and penicillin (Life Technologies, Carlsbad, USA) (1%) and incubated overnight at 37°C in 5% CO_2_. A minimum of five replicate wells were plated for each condition. Sensor cartridges were hydrated overnight in Seahorse XF calibrant at 37°C without CO_2_ supplementation. On the day of the assay, macrophages were thoroughly washed with PBS and the culture medium was replaced with XF DMEM supplemented with glutamine (2 mM; Sigma-Aldrich, Saint-Louis, USA), pyruvate (1 mM; Sigma-Aldrich, Saint-Louis, USA), and glucose (10 mM; Sigma-Aldrich, Saint-Louis, USA) for the Mito Stress Test or without glucose for the Glycolysis Stress Test. The plate was then incubated for 1h at 37°C without CO_2_ supplementation while the Seahorse XFe96 Analyzer was calibrated. Mito stress test consisted of the sequential injection of oligomycin (1 µM; Sigma-Aldrich, Saint-Louis, USA), FCCP (1 µM; Sigma-Aldrich, Saint-Louis, USA), and rotenone (1 µM; Sigma-Aldrich, Saint-Louis, USA) + antimycin A (1 µM; Sigma-Aldrich, Saint-Louis, USA). Glycolysis stress test was performed using glucose (10 mM), oligomycin (1 µM), and 2-deoxyglucose (100 mM; Sigma-Aldrich, Saint-Louis, USA). Oxygen consumption rates (OCR) and extracellular acidification rate (ECAR) were measured three times with 6 min intervals at the basal level then after each injection. All measures were normalized to the number of DAPI-stained nuclei per well at the end of the experiment, using the Operetta High Content Imaging System (PerkinElmer, Waltham, USA). Cell density, plate coating and FCCP concentration were determined by a dedicated pilot experiment. Data were analyzed with the Agilent Wave desktop software.

### 2.11 *In vitro* study of phagocytosis

To evaluate phagocytosis of *E. Coli*, AT or BM-macrophages were incubated with pH-rodo *E. coli* Bioparticles (100 µg/ml) (Invitrogen, Thermo Scientific, Waltham, USA) in S3 IncuCyte (sartorius, Göttingen, Germany). Pictures of bright field and red fluorescence were acquired each 15 min during 48h. Data were analyzed with S3 IncuCyte software measuring red object intensity on cells area. Areas under the curve were then calculated.

*Candida albicans* strain (isolated from a blood culture of a Toulouse-Rangueil Hospital patient (28)) was seeded on Sabouraud Petri dish 24h before experiment. AT- or BM-macrophages were washed with DMEM medium to remove serum and incubated with *C. albicans* for 30 min at 37°C (ratio 3 yeasts per macrophages). Unbound yeasts were removed by successive washes. Macrophages were then incubated for 4h at 37°C with 5% CO_2_ to evaluate killing of *C. Albicans* or stored at 4°C to evaluate binding of *C. Albicans*. Cells were then lysed by adding sterile H_2_O 5 minutes at room temperature and PBS was added to stop the reaction. Cell lysates were seeded on Sabouraud dextrose agar medium (Bio-Rad, Hercules, USA) and incubated for 24h at 37°C to determine yeast colony forming unit (CFU).

### 2.12 *In vitro* ROS production

AT- or BM-macrophages ROS production was evaluated by adding 5-amino-2,3-dihydro-1,4-phthalazinedione (Luminol; Sigma-Aldrich, Saint-Louis, USA) on culture dishes. Basal chemiluminescence reflecting ROS production was measured continuously during 30 min at 37°C with luminometer ENVISION (PerkinElmer, Waltham, USA).

### 2.13 Single-cell RNA-seq analysis

Sc-AT single cell dataset was obtained from Emont et al. (29) (#GSE176171) (GSM5820690_Mm_ING_08–3). An initial quality control filtering was performed. First, we identified potential single-cell doublets using the Doublet Detection Python package. Further low-quality single cells containing < 200 expressed genes and > 0.1% mitochondrial transcripts, as well as less than 2,000 counts and more than 50,000 counts were excluded from the analysis. Following the removal of low quality and doublet cells, bioinformatics processing of the single cell RNA-seq data was performed using Single Cell Analysis in Python (Scanpy) toolkit. Single cells data were log-transformed and normalized. We applied principal components analyses to reduce the dimensionality of the data. The top 100 principal components selected using the Elbow plot method were used for further analyses analysis. We selected macrophages based on Cd45/*Ptprc*, F4/80/*Adgre1* and Cd11b/*Itgam* and performed an unsupervised agglomerative clustering represented on a UMAP.

### 2.14 Statistics

Comparisons between groups were made using paired or unpaired t-tests, one sample t-test or one-way ANOVA. Prism v10.1.2 software (GraphPad, Dotmatics, Boston, USA) was used for the statistical analyses. p values < 0.05 were considered statistically significant.

For cytokine production, results were analyzed with principal components analysis (PCA) as an unsupervised dimension reduction method. R software 4.2.3 with the factoextra and factomineR packages.

## 3 Results

### 3.1 Generation of AT-macrophages from 3D culture system

**Figure 1:**
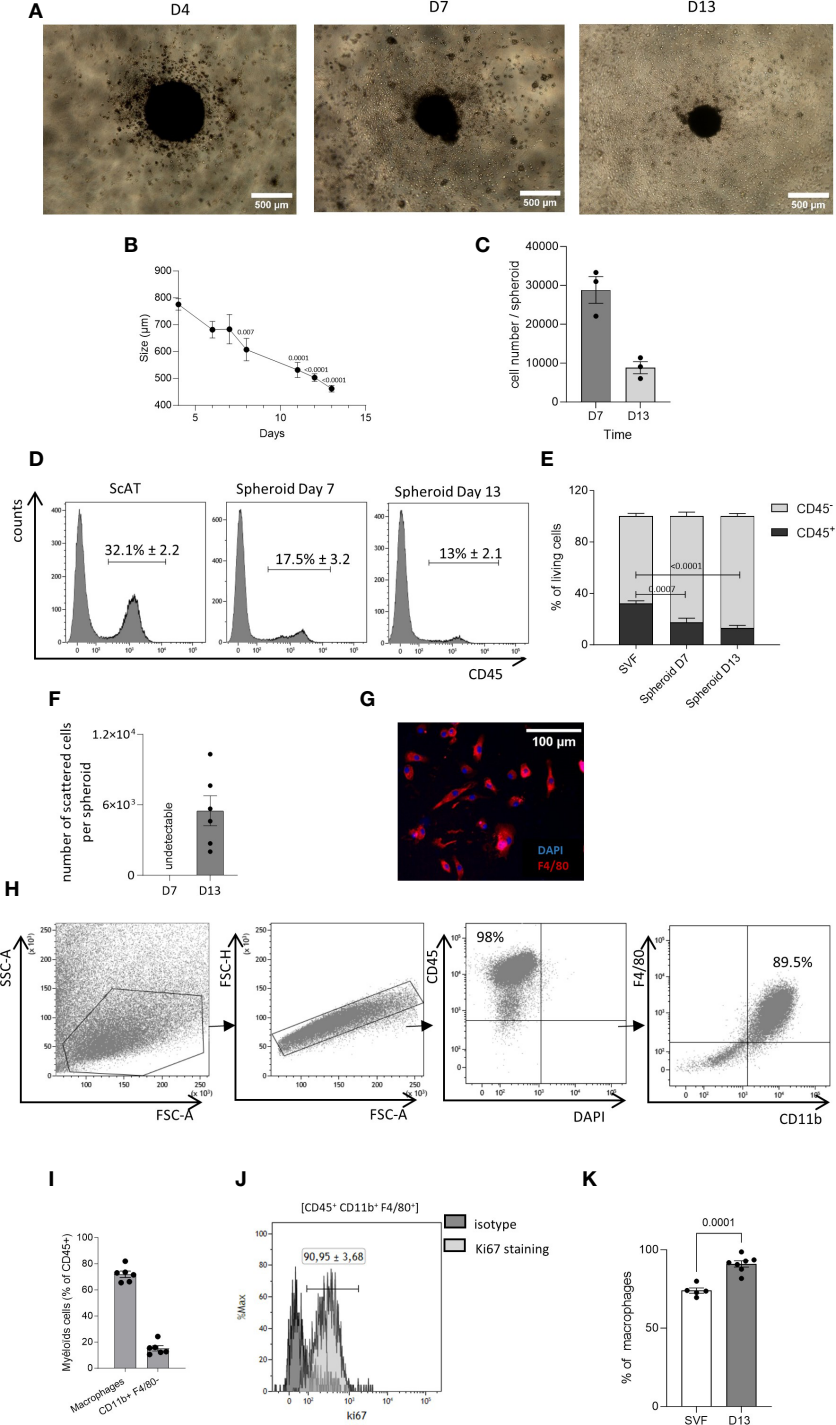
Generation of AT-macrophages from 3D culture system. The seeding of the stromal vascular fraction (SVF) cells obtained from the murine sc–adipose tissue (AT) into plates with low adherence leads to the formation of spheroids. **(A)** Representative picture of these spheroids at 4, 7, and 13 days of culture. Acquisition was performed using an inverted Nikon Objective ×40 (scale bar = 500 µm). **(B)** Kinetic of the spheroid size. *P*-values were obtained by comparing the mean values of each time point with the mean value of day 4. **(C)** Spheroid cell number at days 7 and 13 of culture. (**D, E**) After dissociation, at days 7 and 13 spheroid composition was analyzed by flow cytometry. **(D)** Representative histograms of CD45 expression in dissociated cells from scAT SVF, spheroid day 7 and spheroid day 13, gated on singlets DAPI^−^ (4’,6-diamidino-2-phenylindole) cells. **(E)** Quantification of hematopoietic (CD45^+^) and non-hematopoietic (CD45^−^) cells in the scAT SVF and within the spheroid, expressed in % of DAPI^−^ cells. **(F–K)** Starting at day 10 of culture, dispersed cells migrate outside of the spheroids. **(F)** Number of scattered cells per spheroid obtained after 7 and 13 days in culture. **(G)** Representative picture of cells that have spread out of the spheroid after 13 days in culture (scale bar = 100 µm). Macrophages were stained with F4/80 (red) and their nuclei stained with DAPI (blue). **(H)**. Representative dot plot of flow cytometry showing the expression of macrophage-specific markers on the surface of scattered cells out of the spheroid and selected among singlet DAPI^−^ cells. **(I)**. Percentage of macrophages (CD45^+^/F4/80^+^/CD11b^+^) and other myeloid cells (CD45^+^/CD11b^+^/F480^−^) among CD45^+^ scattered cells surrounding spheroids and quantified by flow cytometry at day 13. **(J)** Representative histogram of flow cytometry showing the expression of Ki67 on AT cultured macrophages outside spheroids. **(K)** Quantification of Ki67 staining in the SVF and AT cultured macrophages outside spheroids. Results are expressed as mean ± SEM and compared using one-way ANOVA and *t*-test.

We adapted a previously published protocol of 3D culture, used to generate AT-mast cells (23). The cells of stroma vascular fraction (SVF) derived from the sc-AT were cultured on ultra-low adherent plates with medium supplemented with M-CSF. This resulted in the development of spheroids within 4 days of culture initiation. Spheroids were then maintained for at least 13 days (**Figure 1A**). The size of the spheroids (**Figure 1B**), and their cell content (**Figure 1C**) decreased from 4 to 13 days in culture. Spheroids cell composition was analyzed by flow cytometry after enzymatic dissociation and compared to sc-AT composition. Spheroids contained mainly stromal cells (CD45^−^), and 10% to 20% of immune cells (CD45^+^) (**Figures 1D, E**). The analysis of the stromal population revealed that the spheroid contained a high percentage (80%) of cells expressing mesenchymal markers (CD90, CD29), including CD24^+^ cells, (**Supplementary Figures S1A–D**). In contrast, the proportion of endothelial cells identified as CD45^-^/CD31^+^ was significantly lower in the spheroid compared to the SVF, indicating that they were not maintained in the 3D cultures (**Supplementary Figures S1E, F**).

In addition, based on the phenotypes described in the BM and in the sc-AT (30, 31), hematopoietic stem/progenitor cells were identified in the spheroid over time (**Supplementary Figure S2A**). Hematopoietic stem/progenitor cells population (LSK: Lin^-^/Sca1^+^/CD117^+^) was enriched in the spheroid compared to uncultured SVF (**Supplementary Figure S2B**). The proportion of engaged myeloid progenitors including Megakaryocyte-Erythrocyte Progenitor (Lin^−^/CD117^+^/Sca-1^−^/CD16/32^−^/CD34^−^), common myeloid progenitor (Lin^−^/Sca1^−^/CD117^+^/CD16/32^−^/CD34^+^) and Granulocyte-Macrophage Progenitor (Lin^−^/Sca1^−^/CD117^+^/CD16/32^+^/CD34^+^) was less than 1% of the total living cells in the spheroid. These percentages were comparable to those observed in uncultured SVF (**Supplementary Figure S2C**).

The analysis of the immune mature population showed that the proportion of CD45^+^ cells decreased over time, and was significantly lower than the percentage of immune cells present in the uncultured SVF under homeostatic conditions (**Figures 1D, E**). Both myeloid (CD45^+^/CD11b^+^) and lymphoid (CD45^+^/CD11b^−^) cells were identified in the spheroid as in the sc-AT SVF (**Supplementary Figure S3A**). Lymphoid cells accounted for 3%–5% of the SVF or the spheroid (**Supplementary Figures S3A, B**). In contrast, the percentage of myeloid cells was significantly lower in the spheroid compared to the SVF and decreased over time in culture, suggesting that myeloid cells did not persist within the spheroid (**Supplementary Figure S3B**). To further analyze the myeloid population, we examined monocytes (CD45^+^/CD11b^+^/F4/80^−^/Ly6C^+^), dendritic cells (CD45^+^/CD11b^+^/F4/80^−^/CD11c^+^) and neutrophils (CD45^+^/CD11b^+^/F4/80^−^/Ly6G^+^), present in the sc-AT SVF. We found that these cell types were almost absent from the spheroid (**Supplementary Figures S3C–H**). In contrast, macrophages (CD45^+^/CD11b^+^/F4/80^+^/Ly6C^−^) accounted for 50% of the immune population in both the sc-AT SVF and the spheroid at day 7, but this percentage decreased significantly by day 13 of culture (**Supplementary Figures S3C, D**). This reduction is concomitant to a significant decrease in the percentage of proliferating macrophages contained in the spheroid after 13 days of culture (**Supplementary Figure S3I**). Overall, spheroids represent a three-dimensional structure composed of various cell populations present in the sc-AT and preserved over time.

Starting from day 10 of culture, dispersed cells emerged surrounding the spheroid and at day 13, up to 5 × 10^3^ cell/spheroid were obtained (**Figure 1F**). These cells formed a homogeneous immune population mostly expressing the macrophage cell surface markers CD45, CD11b, and F4/80, as shown by flow cytometry and immunohistochemistry (**Figures 1G, H**). Macrophage purity reached 71.98 ± 2.42% after 13 days in culture (**Figure 1I**). The 3D culture system also generated CD11b^+^/F4/80^−^ myeloid cells, accounting for 15.21 ± 2% of the population surrounding the spheroid (**Figure 1I**). Flow cytometry analyses also showed that the large majority of AT-cultured macrophages expressed Ki67 (**Figures 1J, K**), suggesting that macrophages were proliferating in these conditions. Hence, this approach generates living tissue macrophages in culture without the need for purification by cell sorting.

### 3.2 Cultured AT-macrophage population exhibits specific features compared to macrophages derived from medullar monocytes

**Figure 2:**
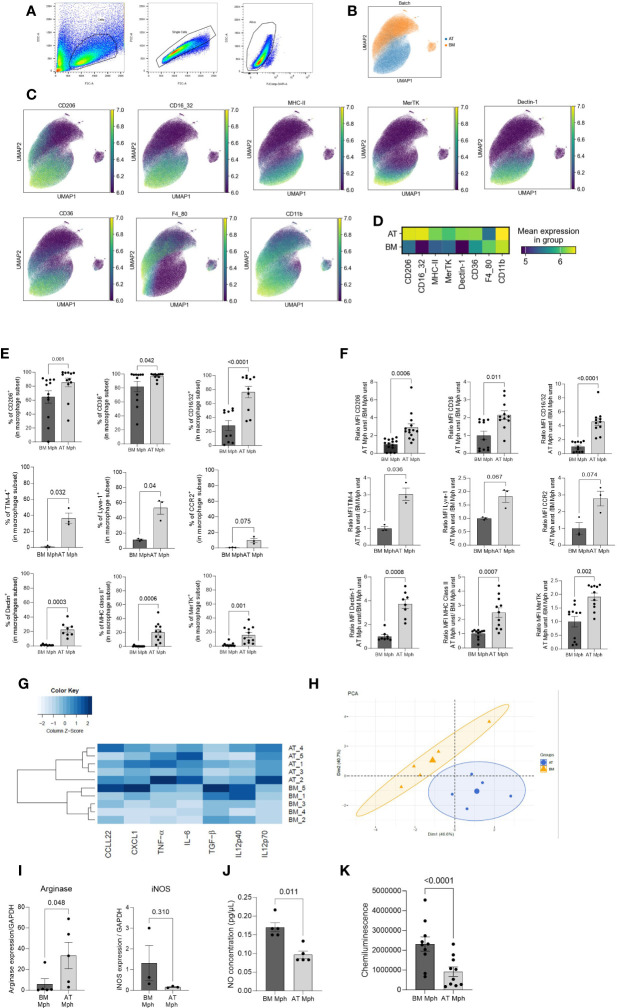
AT-macrophages are mature and phenotypically different from BM-macrophages. Macrophages generated from adipose tissue (AT) spheroids or from bone marrow (BM) monocyte differentiation were harvested and then seeded on adherent culture dishes, for 24h. Cells and supernatant were then collected and analyzed by flow cytometry. **(A)** Gating strategies for AT- and BM-macrophages. **(B–D)** Unbiased analysis of 5 independent samples of AT- and BM-macrophages realized using Uniform Manifold Approximation and Projection (UMAP) visualization with the signal strength of key phenotypic macrophages markers. **(B)** Cell clustering of both macrophage populations showing that AT- and BM-macrophages are distinct populations. **(C)** Protein expression patterns projected of CD206, CD16/32, MHC-II, MerTK, Dectin-1, CD36, F4/80, and CD11b MFI represented on a UMAP and illustrated using blue-green-yellow continuous color scale. **(D)** Heatmap visualization of surface markers expression on AT- and BM-macrophages. **(E)** Percentage of AT- and BM-macrophage (CD45^+^/CD11b^+^/F4/80^+^) expressing extracellular markers: CD206, CD36, CD16/32, Dectin-1, MHC-II, MerTK, TIM-4, Lyve-1, CCR2, (*n* = 3 to 14). Results are expressed as percentage of macrophage population and compared using paired *t*-test. **(F)**. MFI of macrophages markers analyzed by flow cytometry in AT- and BM-macrophages (*n* = 3 to 14). Results are expressed as ratio of values obtained in BM-macrophages and compared using paired *t*-test. **(G)** Heatmap performed on the production of seven cytokines (pg/µl) quantified by LEGENDplex in supernatant of AT- and BM-macrophages cultures in unstimulated conditions. The dendrogram performed using complete linkage method was able to cluster BM- and AT-macrophages on the basis of their cytokines production. (*n* = 5). **(H)** Principal components analysis (PCA) performed on macrophages cytokine production. BM- and AT-macrophages were colored in orange and blue, respectively. **(I)** *Arginase-1* and *iNOS* mRNA relative expression evaluated by RT-qPCR (*n* = 3–5). Results are express as a ratio of housekeeper gene expression (GAPDH). **(J)** Nitric Oxyde (NO) production was quantified in the culture medium using Griess Reagent (*n* = 5). Results are expressed in pg/µl. **(K)** ROS production was followed during 90 min using Luminol and Chemiluminescence was measured (*n* = 10). Results were compared using paired *t*-test.

Macrophages generated from AT spheroids were compared to macrophages obtained through classical medullar monocyte differentiation. To this end, both macrophage populations were harvested and seeded on adherent culture dishes, for 24h before flow cytometry analysis. Singlet and viability gating was performed manually prior to data export for analysis (**Figure 2A**). Individual flow cytometry standard files of CD45^+^ cells in each type of culture were concatenated to ensure spatial alignment of the same population between samples. A total of 17,409 cells were randomly used per sample to create a final file containing 15,6681 total cells. Then macrophages were selected on the basis of CD11b and F4/80 expression. UMAP dimensional reduction revealed a clear difference between AT- and BM-macrophages (**Figure 2B**). Protein expression patterns of selected antigens (CD206, CD16/32, MHC-II, MerTK, Dectin-1, and CD36), classically used to characterize macrophage subsets, were superimposed on a UMAP (**Figure 2C**) and highlighted on a heatmap (**Figure 2D**), revealing that most of these markers were differentially expressed by AT- and BM-macrophages that thus clustered separately.

The comparison between both populations was then quantified using conventional flow cytometry analysis. The percentage of positive cells (**Figure 2E**) and the MFI (**Figure 2F**) were shown for classical core macrophage surface markers. AT-macrophage population was highly homogeneous regarding the content of CD206, CD36, and CD16/32 positive cells, that represented 75%–97% of the population. TIM4 and Lyve-1, 2 cell surface markers specific for resident macrophages, were expressed by 36%–53% of AT-macrophages. CCR2, Dectin, MHC-II, and MerTK were only detected on 9%–20% of the AT-macrophages. The percentage of positive cells for all these cell surface markers was significantly higher in the AT-macrophages than in the BM-macrophages except for CCR2 (**Figure 2E**). According to the UMAP visualization (**Figure 2C**), AT-macrophages showed a significantly higher MFI for all these markers except CCR2 and Lyve 1 in comparison to BM-macrophages (**Figure 2F**).

In order to evaluate the functional capacity of AT- and BM-macrophages, the production of cytokines was quantified in the cell culture supernatants in unstimulated conditions. We used LEGENDPlex bead-based assays to quantify the amount of secreted CXCL1, IL-6, TNF-α, IL12p40, IL12p70, TGF-β, and CCL22 via flow cytometry. AT- and BM-macrophages exhibited specific cytokine production signature (**Figure 2G**). This clear segregation appears to be due to a higher secretion of CXCL1, IL-6, and CCL22 by AT-macrophages compared to BM-macrophages (**Supplementary Figure S4**). These results were confirmed through PCA (**Figure 2H**), which demonstrated a clear distinction in cytokine production profiles between AT- and BM-macrophages. We performed RT-qPCR to assess the expression of *iNOS and Arginase-1*, as representative pro- and anti-inflammatory polarization markers respectively (**Figure 2I**). Unstimulated AT-macrophages expressed *Arginase-1* while BM-macrophages expressed *iNOS*. Consequently, AT-macrophages generated lower levels of NO and ROS than BM-macrophages (**Figures 2J, K**), indicating that in the absence of stimulation, AT-macrophages exhibit a less pro-inflammatory phenotype than BM-macrophages. Altogether, these results show that AT- and BM-macrophages have distinct phenotypic and functional characteristics, and suggest that these two populations display different metabolic activities.

### 3.3 Cultured AT-macrophages display high metabolic rate

**Figure 3:**
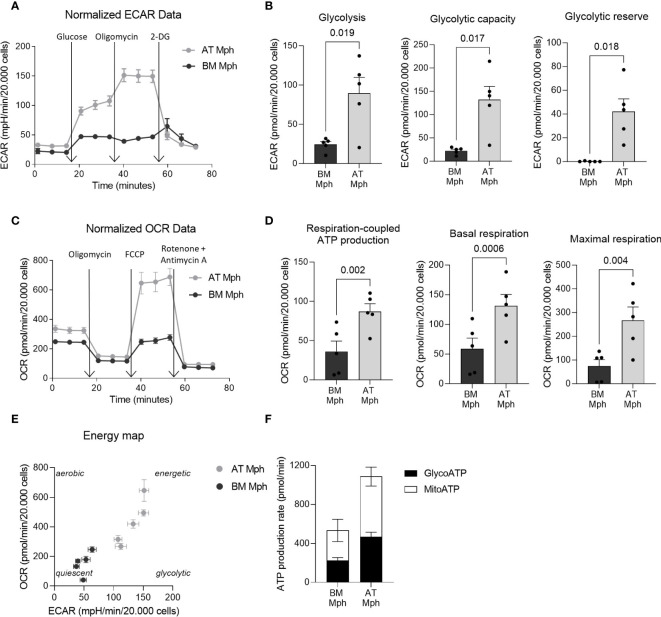
AT-macrophages are more metabolically active than BM-macrophages. Macrophages generated from adipose tissue (AT) spheroids or from bone marrow (BM) monocyte differentiation were seeded onto seahorse plates for 24h before to analyze mitochondrial respiration and glycolytic capacity. **(A–D)** Mitochondrial respiration and glycolytic capacity of AT- and BM-macrophages quantified using Seahorse Agilent Technology 96eXF. Results were normalized to the cell number using DAPI staining after the assay. **(A, B)** Extracellular acidification rate (ECAR) upon glycolytic stress (injection of glucose, oligomycin, and 2-deoxyglucose) was measured. **(A)** Representation of real-time measurement of ECAR. **(B)** Glycolysis, glycolytic capacity and glycolytic reserve were calculated (*n* = 5). **(C, D)** Oxygen consumption rate (OCR) upon mitochondrial stress (injection of oligomycin, FCCP, rotenone and antimycin A) was measured. **(C)** Representation of real-time measurement of OCR. **(D)** Basal respiration, maximal respiration and respiration-coupled ATP production were calculated (*n* = 5). **(E)** Energy map of maximal respiration versus glycolytic capacity after FCCP injection (*n* = 5). **(F)** Glyco- and mito-ATP production rates were calculated using the aforementioned ECAR and OCR data (*n* = 5). Results were compared using paired *t*-test.

Specific metabolic pathways are increasingly being recognized as essential hallmarks of macrophage subsets. We thus compared the energetic metabolism of AT- and BM-macrophages using the Seahorse technology. This allows the real-time measurement of ECAR and OCR as indicators of glycolytic function and oxidative phosphorylation, respectively, as readouts of cellular bioenergetic profiles in response to metabolic stressors. Glycolysis stress tests were conducted to evaluate the glycolytic function of macrophages. The ECAR of AT-macrophages was higher than that of BM-macrophages (**Figure 3A**), leading to a significant increase in key glycolytic function parameters such as glycolysis (3.7-fold), glycolytic capacity (5.9-fold), and glycolytic reserve (300-fold) (**Figure 3B**). To determine whether BM-macrophages exhibit compensatory mitochondrial metabolism, mitochondrial stress tests were performed. Interestingly, AT-macrophages exhibit higher OCR compared to their BM counterparts (**Figure 3C**). This was highlighted by a significant increase in basal respiration (2.2-fold), maximal respiration (3.6-fold) and respiration-coupled ATP production (2.4-fold) (**Figure 3D**). These results indicate that AT-macrophages exhibit a highly energetic metabolic state (high glycolytic capacity and concomitant respiration) compared to BM-macrophages that adopt a relatively low bioenergetic profile, reminiscent of a quiescent state (**Figure 3E**). Additionally, the increased metabolic activity in AT-macrophages resulted in a higher rate of ATP production, which was twice that of BM-macrophages (**Figure 3F**).

### 3.4 Pro/anti-inflammatory polarization induction of cultured AT-macrophages

**Figure 4:**
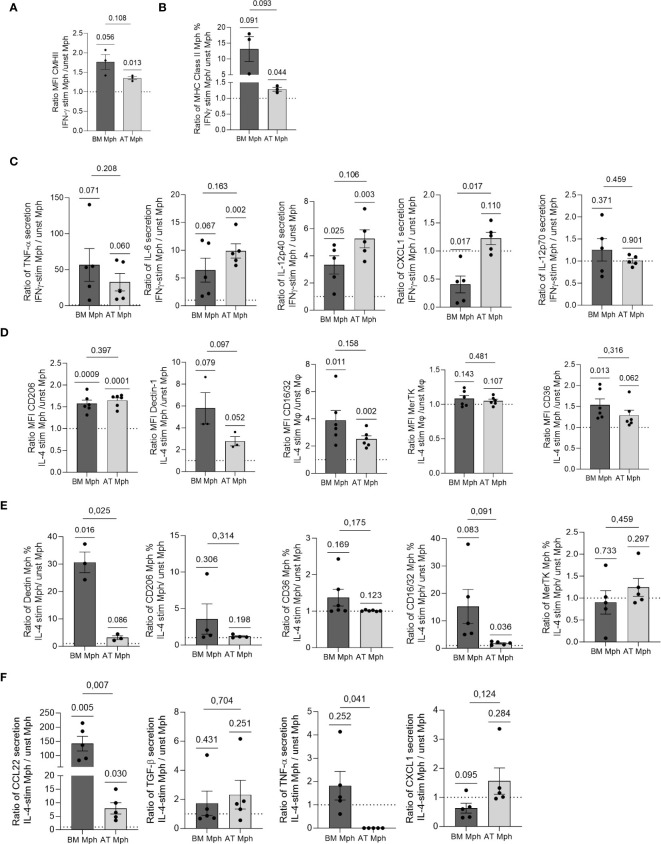
AT- and BM-macrophages are differentially polarized by IL-4 or IFN-γ treatment. Macrophages generated from adipose tissue (AT) spheroids or from bone marrow (BM) monocyte differentiation were seeded onto adherent culture dishes for 24h, and treated with IFN-γ **(A–C)** or IL-4 **(D–F)** for another 24h. Flow cytometry was used to assess MFI **(A, D)** and % of positive cells **(B, E)** for each cell surface marker expressed by macrophages after treatment (*n* = 3–6). **(C, F)** Supernatants were collected and cytokine production quantified by LEGENDPlex (*n* = 5) in AT- and BM-macrophages. Results were compared using paired *t*-test. One Sample *t*-test analysis was used to compare stimulated and unstimulated conditions.

We compared the polarization abilities of cultured AT- and BM-macrophages in response to IFN-γ and IL-4. Specifically, we assessed the induction of pro- or anti-inflammatory cell surface markers and the production of corresponding cytokines in the cell culture supernatants using flow cytometry, after 24h of stimulation. The AT-macrophages demonstrated a significant increase in both the MFI and positive cell percentage for MHCII in response to IFN-γ treatment. A comparable response was observed in BM-macrophages although to a lesser extent (**Figures 4A, B**). This increase in MHC-II was associated with increased secretion of TNF-α, IL-6, and IL-12p40 in both types of macrophages (**Figure 4C**). Interestingly, only BM-macrophages treated with IFN-γ showed decreased CXCL1 secretion, while IL-12p70 expression remained unchanged (**Figure 4C**).

IL-4 treatment was used to polarize macrophages towards an anti-inflammatory phenotype. As expected, the treatment resulted in enhanced MFI of CD206, Dectin-1 and CD16/32 in both AT- and BM-macrophages (**Figure 4D**). Although MerTK MFI level remained unchanged, CD36 was induced specifically in BM-macrophages (**Figure 4D**). Except for Dectin-1 in BM-macrophages, IL-4 did not elicit any change in the percentage of positive cells for each cell surface marker (**Figure 4E**). IL-4 treatment significantly increased the production of CCL22 in both AT- and BM-macrophages, with a more pronounced effect observed in BM cultures (**Figure 4F**). While TGF-β secretion was induced to a small extent by IL-4 in AT- and BM-macrophages, the production of TNF-α decreased only in AT-macrophages. Notably, BM-macrophages treated with IL-4 exhibited a reduction in CXCL1 secretion (**Figure 4F**).

Overall, these results showed that AT- and BM-macrophages undergo activation toward a pro-inflammatory phenotype when stimulated by IFN-γ, while the stimulation with IL-4 polarizes them towards an anti-inflammatory phenotype. Notably, while IFN-γ induced comparable changes in AT- and BM-macrophages, AT-macrophages showed less pronounced polarization towards an anti-inflammatory phenotype when stimulated with IL-4, compared to BM-macrophages.

### 3.5 AT- and BM-macrophages show similar phagocytic activity

**Figure 5:**
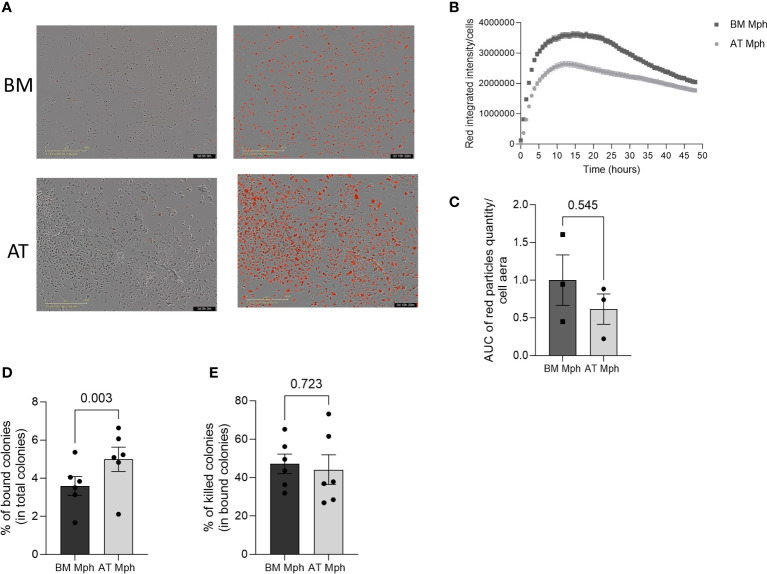
AT- and BM-macrophages show similar phagocytic activity. Phagocytic capacity of *E*. *coli* or *C*. *Albicans* yeast was assessed in adipose tissue (AT)– and bone marrow (BM)–macrophages generated from AT spheroids or from BM monocyte differentiation. **(A–C)** pH-Rodo *E.Coli* were added to macrophages and phagocytosis (apparition of red particles) was analyzed on IncuCyte during 48h. **(A)** Representatives pictures (IncuCyte) of phagocytose by BM- (up) and AT- (down) macrophages before (left) and after (right) *E*. *coli* addition. **(B)** A representative kinetic curve of bacteria phagocytosis by AT- and BM-macrophages. **(C)** Quantification of *E*. *coli* phagocytosis. Results are express in AUC of red particles reported to cell area. (*n* = 3). **(D, E)** *C*. *Albicans* yeast were added to macrophages for 30 min before assessing binding **(D)** and killing capacity **(E)**. Macrophages were lysed and the supernatants were plated on Sabouraud Petri dishes. Twenty-four hours later, colonies were counted. Results are express as percent of bound among total colonies or killed colonies among total bound colonies. (*n* = 6) Results were compared using Paired *t*-test.

The phagocytic capability of AT- and BM-macrophages was then assessed *in vitro* by incubation with bacteria *E. coli* or yeast *C. albicans*. Phagocytosis of *E. coli* is a classical measure for macrophage function. The pH-sensitive Rhodo *E. coli* was used, which produces red fluorescence in acidic pH conditions, thereby indicating the presence of the bacteria within the phagosome (**Figure 5A**). A rapid and transient increase in red fluorescence was observed in both AT- and BM-macrophages produced *in vitro*, suggesting that all macrophages exhibit rapid phagocytic activity (**Figure 5B**). The phagocytic efficacy of AT- and BM-macrophages towards *E. coli* was found to be comparable (**Figure 5C**). The overexpression of CD206 and Dectin-1 (both being receptors for polysaccharides) in AT-macrophage suggests their potential efficiency in yeast phagocytosis. When incubated with *C. albicans*, AT-macrophages were able to bind and kill the yeasts. Interestingly, AT-macrophages displayed a significantly higher binding capacity than BM-macrophages, although their killing capacity was similar (**Figures 5D, E**).

### 3.6 AT-macrophages produced in 3D culture are similar to sc-AT resident macrophages *in vivo*

**Figure 6:**
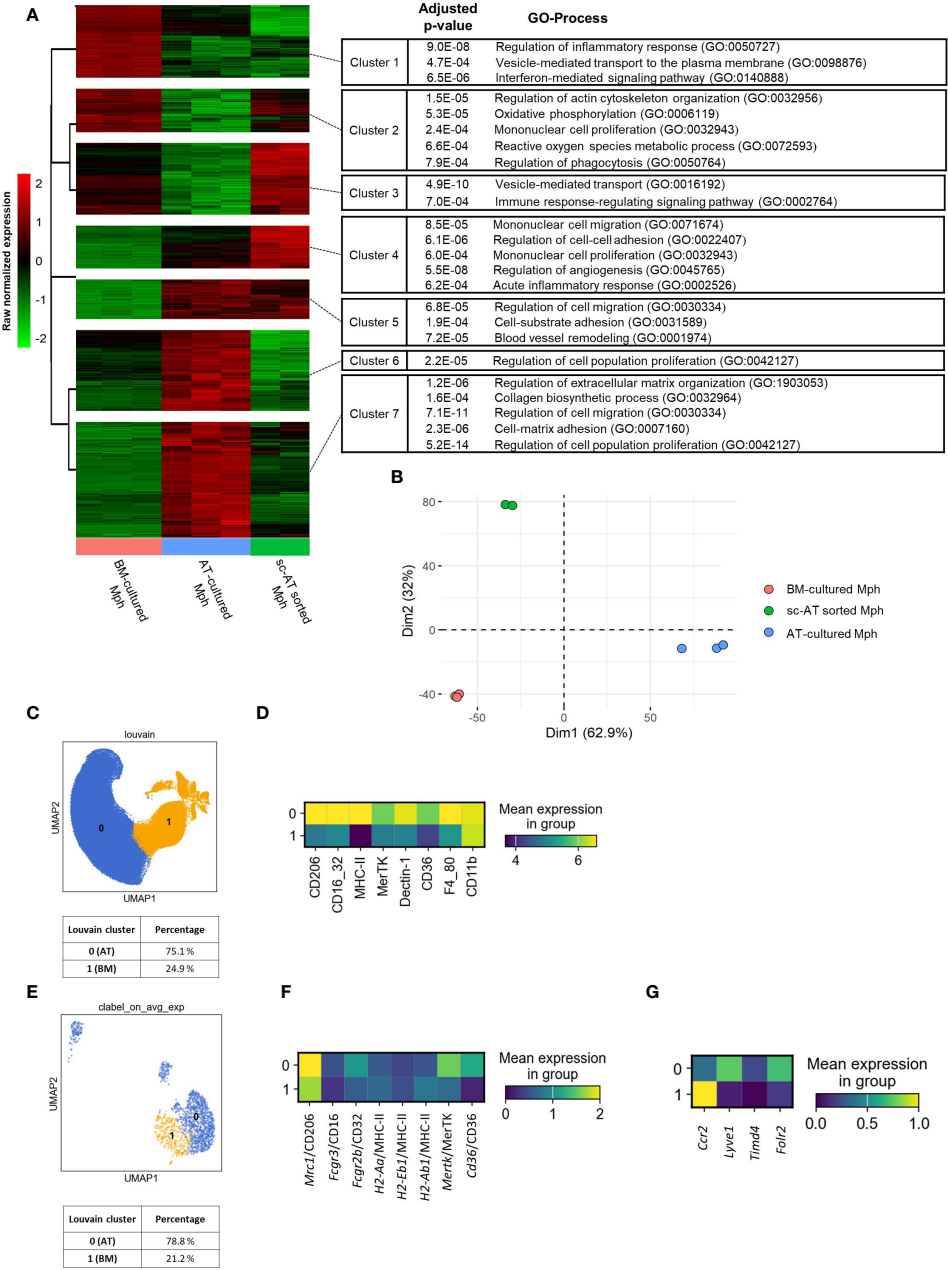
3D cultures to generate macrophages that mirror the phenotypic traits of *in-vivo* AT-resident macrophages. **(A, B)** A bulk RNA-seq analysis was performed on mouse-sorted sc– adipose tissue (AT) macrophages, AT- and bone marrow (BM)–cultured macrophages. **(A)** Hierarchical clustering of the 6,855 genes differentially expressed between AT- and BM-macrophages, according to their expression pattern across all samples, using multiClust R library. GO processes and adjusted *P*-values are mentioned. **(B)**. Principal components analysis (PCA) on standardized gene expression of AT- (blue), BM- (orange), and sc-AT sorted (green) macrophages. **(C, D)** Unbiased analysis of SVF cells obtained from mouse sc-AT was realized by flow cytometry. **(C)**. UMAP showing the clustering (Louvain method) of sc-AT macrophages in two populations and their percentages. **(D)** Heatmap of specific cell surface markers expression of sc-AT macrophages (clusters 0 and 1) (*n* = 7). **(E–G)** Single-cell RNA-seq analysis of sc-AT macrophages extracted from Emont et al. dataset (#GSE176171) (GSM5820690_Mm_ING_08–3) (29). **(E)** UMAP showing agglomerative clustering of macrophage population based on the gene expression. **(F)** Heatmap visualization of *Mrc1*, *Fcgr3*, *Fcgr2b*, *Mertk*, *H2-Aa*, *H2-Eb1*, *H2-Ab1*, and *Cd36* gene expression (for clusters 0 and 1). **(G)**. Heatmap visualization of *Ccr2*, *Lyve1*, *Timd4* and *Folr2* (for clusters 0 and 1).

We then conducted a comparative analysis between the AT-macrophages derived from 3D cultures and *in vivo* sc-AT macrophage populations. To this end, bulk RNA-seq analysis was performed between these two populations, and compared to BM- cultured macrophages. We first removed the culture effect performing a comparative analysis between AT- and BM-cultured macrophages. We identified 6,855 differentially expressed genes (adjusted *p*-value < 0.001) corresponding to specific signatures of AT- and BM- cultured macrophages. To arrange these genes according to the similarity of the gene expression pattern between our three conditions (AT- and BM-cultured macrophages and sc-AT–sorted macrophages), a hierarchical clustering was performed with a beforehand determined optimal number of clusters of 7. **Figure 6A** shows the change in expression of these 6,855 genes between the three conditions, with minimal variations between technical replicates. AT-cultured macrophages and sc-AT sorted macrophages displayed similar pattern of gene expression in three out of seven clusters (clusters numbers 1, 4, and 5). ORA using Gene Ontology biological processes (GO BP) showed that cluster 1 contained genes involved in regulation of inflammatory response, secretion, and interferon-mediated signaling pathways. Genes present in clusters 4 and 5 were mainly involved in cell migration, cell–cell, and cell-substrate adhesion and proliferation (**Figure 6A**). As expected, BM-cultured macrophages also shared similarities with sc-AT sorted macrophages in four out of seven clusters (numbers 2, 3, 6, and 7). These clusters include genes involved in immune response (metabolic process, phagocytosis, secretion, signaling pathway), and cell behavior (proliferation, extracellular matrix organization and biosynthesis, cell migration, and cell-matrix adhesion) (**Figure 6A**). This analysis also showed that genes associated with inflammatory responses exhibited reduced expression levels, whereas genes involved in cell migration and cell adhesion exhibited elevated expression levels in AT- versus BM-cultured macrophages. Two components of a PCA were sufficient to capture 95% of the variance (**Figure 6B**; **Supplementary Figure S5A**). The PCA plot showed a clustering of the conditions, with the first dimension able to discriminate between the AT-cultured macrophage phenotype and the other populations, and the second dimension explaining the BM-cultured macrophage phenotype (**Figure 6B**; **Supplementary Figure S5A**). In order to provide a semantic summary of the genes implicated in each dimension, GSEA was performed for each dimension, utilizing the PCA contribution as a ranking function for GO BP exploration. Among the significant BP with *P* < 0.001, extracellular matrix organization and morphogenesis were enriched in the first dimension, and angiogenesis in the second dimension (**Supplementary Figure S5B**). We then selected the top 5 contributing genes for these 3 BP to plot normalized expression of these genes on an heatmap (**Supplementary Figure S5C**). Using these five genes in each BP, we confirm that these BP discriminate the different macrophage populations as seen in the PCA.

To go further, sc-AT SVF was analyzed using flow cytometry. The data set was represented on a UMAP, a Louvain clustering grouped cells into two distinct clusters (0 and 1), and the percentage of each population was calculated (**Figure 6C**). The expression of macrophage surface markers was represented on a heatmap (**Figure 6D**) and plotted onto a UMAP (**Supplementary Figure S6A**). The clustering of *in-vivo* sc-AT-macrophages revealed a major population (Cluster 0, 75.1%) that highly expressed most of the cell surface markers and a smaller population (Cluster 1, 24.9%) that also expressed these markers but at a lower level (**Figure 6D**). Cluster 0 of *in-vivo* sc-AT-macrophages displayed a similar profile to AT-macrophages obtained in culture (**Figures 2C, D**), as revealed by heatmaps comparison. In contrast, cluster 1 of *in-vivo* sc-AT macrophages resembled BM-macrophages generated in culture (**Figures 2C, D**). To go further, we explored single-cell gene expression of sc-AT macrophage populations obtained from Emont et al’s dataset (29) (**Figures 6E–G**). Agglomerative clustering revealed two distinct populations: clusters 0 and 1 accounting for 78.8% and 21.2% of the total population, respectively (**Figure 6E**). Both heatmap and UMAP plots were used to display the expression of genes coding for the cell surface markers (*Mrc1* as CD206; *Fcgr3* and *Fcgr2b* as CD16/32; *Cd36* as CD36; *Mertk* as MerTK; *H2-Aa*, *H2-Eb1* and *H2-Ab1* as MHC-II) (**Figure 6F**; **Supplementary Figure S6B**). In accordance to the flow cytometry results, this analysis showed that cluster 0 displayed exclusive gene expression compared to cluster 1 (**Figure 6F**; **Supplementary Figure S6B**). These results indicated that the AT-macrophages produced in 3D cultures have comparable phenotypic and genotypic characteristics to one of the two macrophage populations described in the sc-AT *in vivo* (cluster 0). Cluster 1, on the other hand, closely resembles BM-macrophages.

Strikingly, in single-cell analysis of the sc-AT *in vivo*, cluster 0 (similar to AT-cultured macrophages) showed high expression of Lyve1 and Folr2 and low expression of Timd4 (**Figure 6G**; **Supplementary Figure S6C**) which have been described as a gene signature for resident macrophages (10). Conversely, cluster 1 (resembling BM-macrophages) showed high expression of Ccr2 (**Figure 6G**; **Supplementary Figure S6C**), which is recognized as a signature of monocyte-derived macrophages infiltrating tissue. These findings suggest that macrophages produced in AT-3D cultures mimic the resident macrophage population found *in vivo.*

## 4 Discussion

AT-macrophages are essential in both regulating AT homeostasis and preventing metabolic diseases (31–36). Our study provides a 3D *in-vitro* system for generating and culturing AT-resident functional macrophages, without the need for cell sorting. This system also paves the way for subsequent functional analyses of these highly specialized cells.

To date, only a limited number of studies have provided a comprehensive description of the isolation and *in-vitro* culture of resident macrophages without modifying their function. Alveolar macrophage-like cells were obtained from BM-monocyte differentiation by adding cytokines in the culture medium (37). Various techniques have been developed to culture resident macrophages, including co-culturing with stromal cells (38, 39). Although these techniques are effective for maintaining and preserving the function of resident macrophages in the long term, they require subsequent separation of the macrophages from the stromal cells for analysis. Unfortunately, this step is often deleterious to the cells. The 3D system we have developed provides an optimal microenvironment for culturing resident macrophages. Indeed, the microenvironment plays a pivotal role in macrophage polarization and survival. Macrophages are continually adapting to their environment in order to effectively eliminate cells, debris, and released molecules and maintain homeostasis (14, 40). All tissues exhibit distinct characteristics and create a unique microenvironment that determines the phenotype and function of the macrophage (41). In this study, spheroids derived from sc-AT SVF preserved cells present in the AT stroma, and mimicked the microenvironment of AT-macrophages. It has to be noted that the spheroids contain native AT-resident macrophages, hematopoietic myeloid precursors, and a few circulating monocytes, all of which may contribute to the generation of macrophages within the 3D culture. Nevertheless, to use this culture system in other contexts or for other tissues is conditioned by the capacity of the cells to form a 3D structure.

The 3D cultures generate cells, which exhibit specific cell surface markers of mature macrophages including CD11b and F4/80. They also display functional hallmarks of mature macrophages, and are capable of responding to pro- and anti-inflammatory stimulation, through adapting their cell surface and secretory phenotype. In addition, these cells exhibit efficient phagocytosis of various pathogens. When compared, AT- and BM-macrophages display distinct characteristics, primarily attributed to specific gene profile, differential expression of macrophage markers, ROS production, and basal respiration. Overall, the phenotypic and metabolic features of AT- and BM-macrophages obtained *in vitro* indicate different basal state polarization, with AT-macrophages exhibiting a more anti-inflammatory phenotype than BM-macrophages, in line with *in-vivo* data on resident versus monocyte-derived macrophages (3, 25). IL-4 treatment elicits a stronger response in BM-macrophages compared to AT-macrophages potentially due to the high expression of anti-inflammatory phenotypic markers in the basal state in these AT-macrophages. In contrast, IFN-γ has a similar effect on both populations, consistent with the same phagocytic capacity. This has already been described for Kupffer cells, which respond similarly to BM-macrophages to an infectious challenge *in vivo* despite a distinct transcriptomic signature (42). The phenotype and the response to stimulations in BM-macrophages in culture suggest that they are probably less mature than AT-macrophages, potentially due to the absence of microenvironment.

It is proposed that *in-vivo*, anti-inflammatory AT-macrophages exhibit high oxidative rate capacity and mitochondrial function (43). In contrast, inflammatory macrophages are considered to be glycolytic (44). However, a recent study suggests that glycolysis is essential for the anti-inflammatory macrophage polarization (45). AT-macrophages generated in 3D culture exhibit high levels of both OCR (oxidative phosphorylation rate) and ECAR (extracellular acidification rate), indicating significant metabolic activity following stimulation and a high degree of metabolic plasticity. This metabolic signature has been previously observed in activated macrophages in the peritoneum (46), and in obese AT (47, 48). The metabolic profile of macrophages is an indicator of macrophage activation and largely explains their function in maintaining tissue homeostasis. A change in the microenvironment impacts macrophage metabolism. In AT, obesity is associated with an accumulation of lipids and an increase in both oxidative and glycolytic metabolisms of resident macrophages resulting in an increase in pro-inflammatory phenotype markers (47, 49).

AT-macrophages produced in 3D cultures were able to proliferate, as described for resident AT-macrophages in lean mice (50). An unsupervised genic and phenotypic comparison of macrophage populations isolated from sc-AT, 3D culture system, or medullary monocyte differentiation showed that macrophages produced in 3D culture correspond to the fraction of resident macrophages present *in vivo* within the AT. Indeed, AT-cultured macrophages and sc-AT-macrophages shared functional similarities identified in bulk RNA-seq analysis, that appear to be involved in the maintenance of the tissue microenvironment (adhesion, migration, vessel remodeling). In addition, they express markers of resident cells such as TIM4 and Lyve1 (10). Moreover, most of the macrophages generated in 3D culture highly express the CD206, a cell surface marker defined by Félix et al. as a hallmark of AT-resident macrophages (11). This cell surface marker has also been reported to identify M2-like AT-resident macrophages, which serve as a niche for adipocyte progenitors in AT (51). Furthermore, these macrophages display minimal expression of CCR2, a marker indicative of macrophages that have migrated to AT (21). In physiological conditions, these macrophages are sparsely distributed within AT (52). Maintaining an appropriate balance of these two macrophage subsets is crucial for tissue homeostasis. Indeed, a disruption of this balance with an accumulation of macrophages derived from circulating monocytes within AT is observed in obese individuals (52). The application of this culture method to pathological AT could provide a more comprehensive understanding of the intrinsic defaults in resident macrophage populations.

In summary, our study yields an efficient culture technique that maintains the phenotypic and functional characteristics of resident AT-macrophages. This method stands as a valuable resource for exploring the differentiation and function of AT-macrophages *in vitro* in diverse physiological and pathological contexts.

## Data availability statement

The original contributions presented in the study are included in the article/**Supplementary Material**. Further inquiries can be directed to the corresponding author.

## Ethics statement

The animal study was approved by European Community Guidelines (2010/63/UE)/institutional ethics committee N.122 US006/CREFRE. The study was conducted in accordance with the local legislation and institutional requirements.

## Author contributions

AA: Conceptualization, Writing – original draft, Writing – review & editing, Formal analysis, Investigation, Methodology. MR: Formal analysis, Investigation, Methodology, Writing – review & editing. JN: Formal analysis, Investigation, Methodology, Writing – review & editing. MT: Formal analysis, Investigation, Methodology, Writing – review & editing. JF: Formal analysis, Investigation, Methodology, Writing – review & editing. EA: Formal analysis, Investigation, Methodology, Writing – review & editing. CD: Resources, Writing – review & editing. HA: Resources, Writing – review & editing. PM: Writing – review & editing, Formal analysis, Investigation, Methodology, Validation. AC: Resources, Writing – review & editing. LC: Resources, Writing – review & editing. MO: Resources, Writing – review & editing, Formal analysis, Investigation, Methodology. BC: Conceptualization, Funding acquisition, Resources, Supervision, Writing – original draft, Writing – review & editing.
