# Supplementary material for: Generation of functionally active resident macrophages from adipose tissue by 3D cultures
Source: Front Immunol. 2024 Jun 21;15:1356397. doi: 10.3389/fimmu.2024.1356397 (PMC11224291; doi:10.3389/fimmu.2024.1356397)
Supplement: Supplementary file 1 [file Presentation_1.pdf]

Supplementary Materials

Generation of functionally active resident macrophages from adipose tissue by 3-D cultures

Adèle Arlat[1], Marie-Laure Renoud[1], Jean Nakhle[1], Miguel Thomas[1], Jessica Fontaine[1], Emmanuelle Arnaud[1], Cédric Dray[1], Hélène Authier[1], Paul Monsarrat[1,2,3], Agnès Coste[1], Louis Casteilla[1], Marielle Ousset[1] and Béatrice Cousin[1*]

[1] RESTORE Research Center, Université de Toulouse, INSERM 1301, CNRS 5070, EFS, ENVT, Toulouse, France

[2] Dental Faculty and Hospital of Toulouse – Toulouse Institute of Oral Medicine and Science, CHU de Toulouse, France.

[3] Artificial and Natural Intelligence Toulouse Institute ANITI, France.

*** Correspondence:**
Béatrice Cousin : beatrice.cousin@inserm.fr

This work is licensed under CC BY 4.0.

To view a copy of this license, visit **http://creativecommons.org/licenses/by/4.0/**

1 Supplementary Figures and Tables

1.1 Supplementary Figures

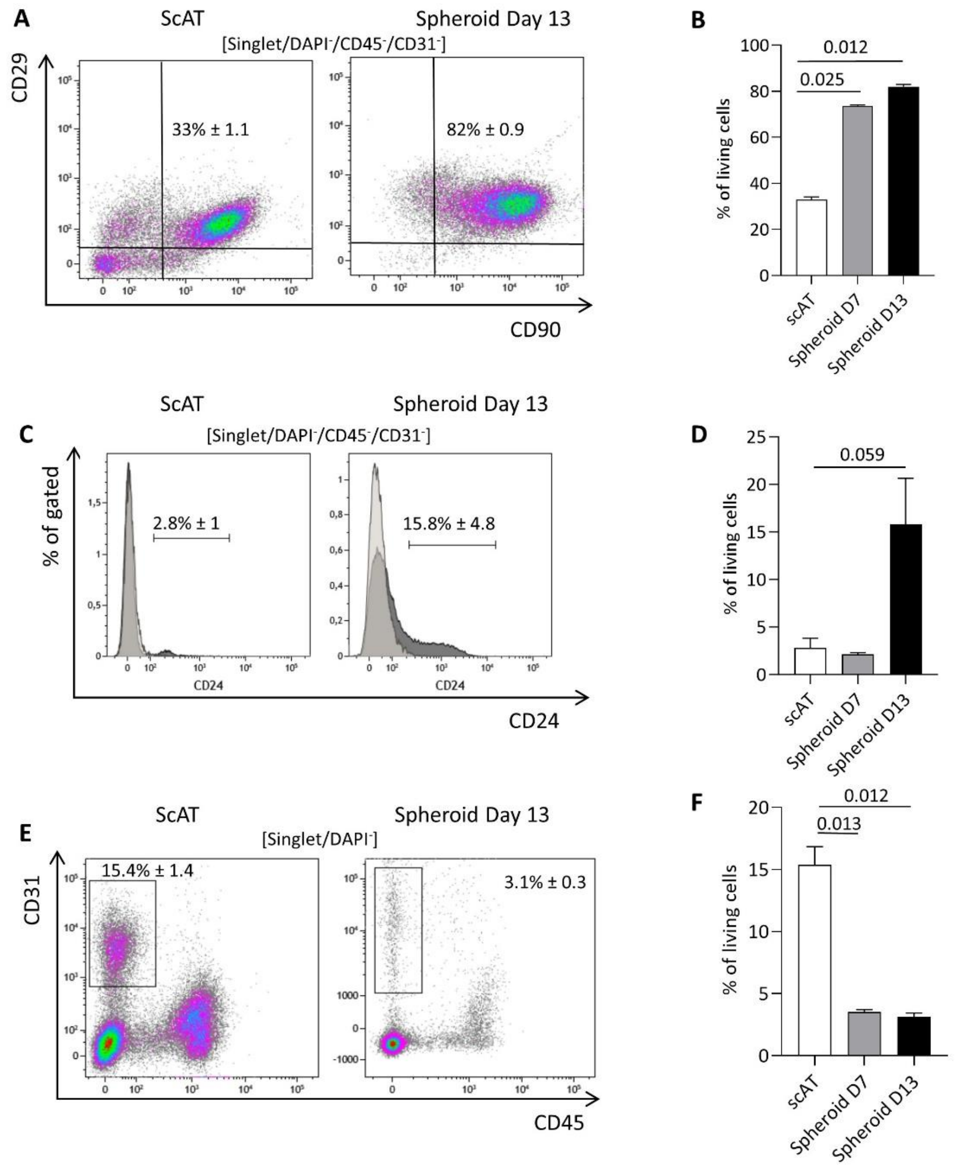

Supplementary Figure 1. Enrichment of the spheroid with stromal cells in comparison to uncultured SVF. The seeding of the SVF cells obtained from the murine sc-AT into plates with low adherence leads to the formation of spheroids. **A.** Representative dot plots of flow cytometry showing the expression of mesenchymal specific markers (CD90, CD29) on the surface of dissociated SVF cells (left) and dissociated spheroid cells at Day 13 (right), gated on Singlet/DAPI$^-$/CD45$^-$/CD31$^-$ cells. **B.** Quantification of CD45$^-$/CD31$^-$/CD90$^+$/CD29$^+$ cells in the SVF and the spheroids at day7 and day 13. Results are expressed in percent of living cells. **C.** Representative histogram overlay of CD24 expression (dark grey) and isotype (light grey) in the CD45$^-$/CD31$^-$ population. **D.** Quantification of CD45$^-$/CD31$^-$/CD24$^+$ cells in the SVF and the spheroids at day7 and day 13. Results are expressed in percent of living cells. **E.** Representative dot plots of flow cytometry showing the expression of endothelial specific marker (CD31) and CD45 on the surface of dissociated SVF cells (left) and dissociated spheroid cells at Day 13 (right), gated on Singlet/DAPI$^-$ cells. **F.** Quantification of CD45$^-$/CD31$^+$ cells in the SVF and the spheroids at day7 and day 13. Results are expressed in percent of living cells. All results are expressed as mean ± SEM and compared using One-Way ANOVA.

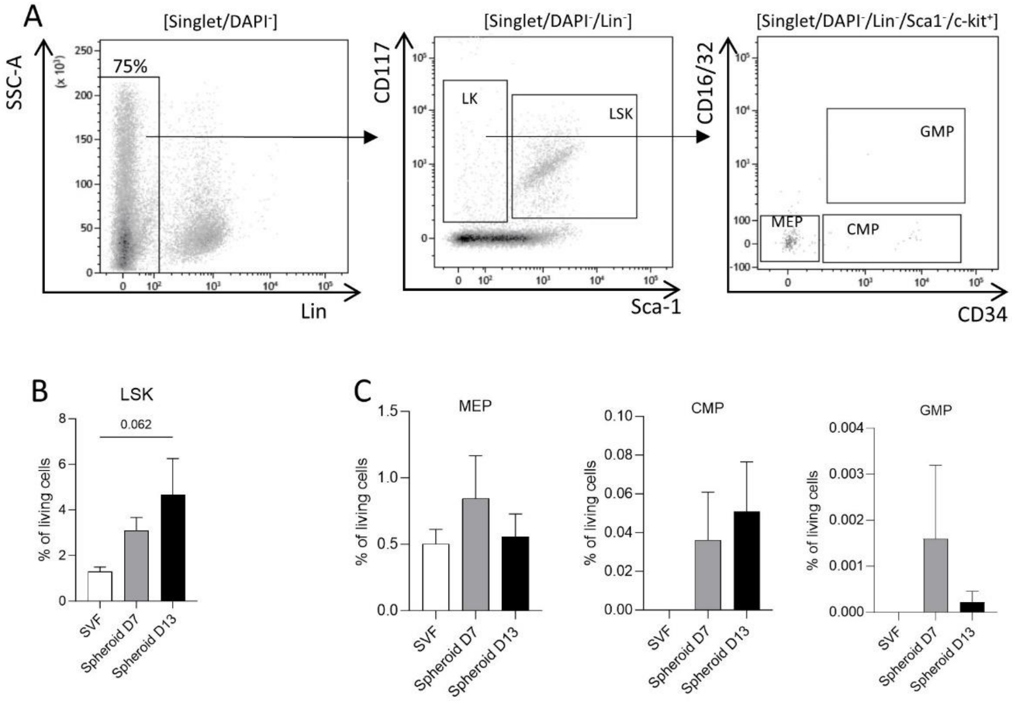

Supplementary Figure 2 : Identification of hematopoietic progenitors in 3D-cultures Flow cytometry analysis of hematopoietic progenitor populations in the SVF cells and dissociated spheroid cells at day 7 and day 13. **A.** Gating strategy of flow cytometry showing the expression of specific progenitor markers (Lin, CD117, Sca-1, CD16/32, CD34) on the surface of dissociated spheroid cells at D13, gated on singlet and DAPI$^-$ cells. **B.** Quantification of LSK (Lin$^-$, CD117$^+$, Sca-1$^+$) cells in DAPI$^-$ population. **C.** Quantification of Megakaryocyte-Erythrocyte Progenitor (MEP; Lin$^-$, CD117$^+$, Sca-1$^-$, CD16/32$^-$, CD34$^-$), Common myeloid progenitor (CMP; Lin$^-$, CD117$^+$, Sca-1$^-$, CD16/32$^-$, CD34$^+$) and Granulocyte-Macrophage Progenitor (GMP; Lin$^-$, CD117$^+$, Sca-1$^-$, CD16/32$^+$, CD34$^+$) cells in SVF cells and dissociated spheroid at D7 and D13. All results are expressed as mean ± SEM and compared using One-Way ANOVA.

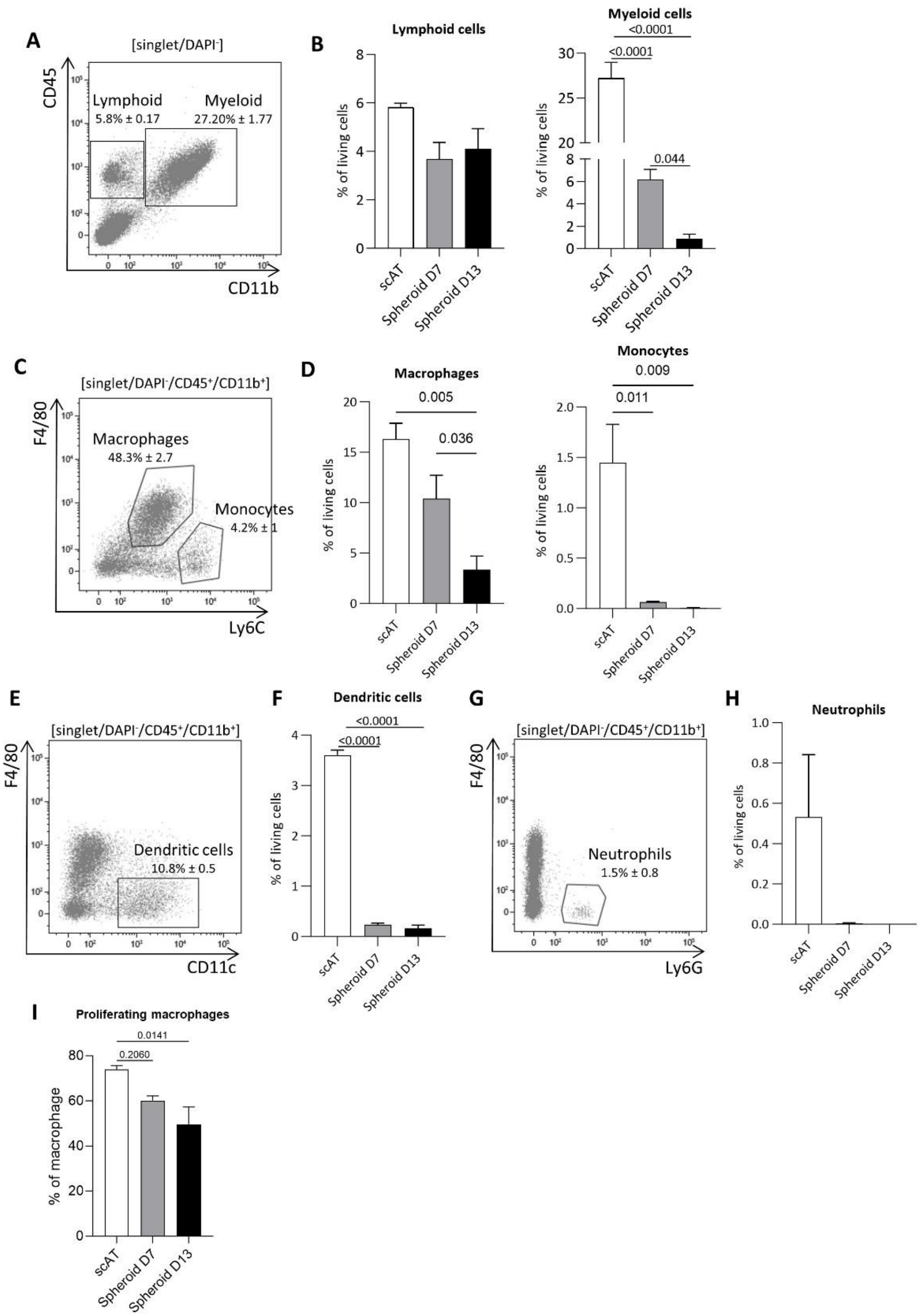

Supplementary Figure 3: Quantification of the myeloid populations in the 3D-cultures **A.** Representative dot plots of flow cytometry showing the expression of myeloid markers (CD11b, CD45) on the surface of dissociated SVF cells, gated on Singlet/DAPI$^-$ cells. **B.** Quantification of CD45$^+$/CD11b$^-$ (lymphoid) and CD45$^+$/CD11b$^+$ (myeloid) cells in the SVF and the spheroids at day7 and day 13. Results are expressed in percent of living cells. **C.** Representative dot plots of flow cytometry showing the expression of monocyte and macrophage markers (F4/80, Ly6C) on the surface

of dissociated SVF cells, gated on Singlet/DAPI$^-$/CD45$^+$/CD11b$^+$cells. **D.** Quantification of CD45$^+$/CD11b$^+$/F4/80$^+$/Ly6C$^-$(macrophages) and CD45$^+$/CD11b$^+$/F4/80$^-$/Ly6C$^+$ (monocytes) cells in the SVF and the spheroids at day7 and day 13. Results are expressed in percent of CD45$^+$ cells. **E.** Representative dot plots of flow cytometry showing the expression of marker of dendritic cells (CD11c) on the surface of dissociated SVF cells, gated on Singlet/DAPI$^-$/CD45$^+$/CD11b$^+$cells. **F.** Quantification of CD45$^+$/CD11b$^+$/F4/80$^-$/CD11c$^+$(dendritic cells) in the SVF and the spheroids at day7 and day 13. Results are expressed in percent of CD45$^+$ cells. **G.** Representative dot plots of flow cytometry showing the expression of marker of neutrophils (Ly6G) on the surface of dissociated SVF cells, gated on Singlet/DAPI$^-$/CD45$^+$/CD11b$^+$cells. **H.** Quantification of CD45$^+$/CD11b$^+$/F4/80$^-$/Ly6G$^+$(neutrophils) in the SVF and the spheroids at day7 and day 13. Results are expressed in percent of CD45$^+$ cells. **I.** Quantification of Ki67$^+$macrophages in the SVF and in the spheroid at day 7 and day 13. Results are expressed in percent of macrophages. All results are expressed as mean ± SEM and compared using One-Way ANOVA.

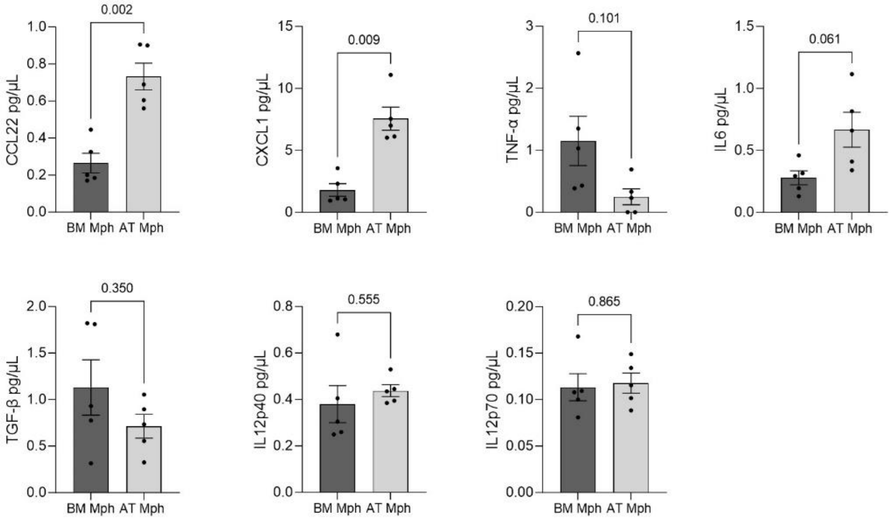

Supplementary Figure 4 : AT and BM cultured macrophages cytokine profile Macrophages generated from AT spheroids or from BM monocyte differentiation were harvested and seeded on adherent culture dishes, for 24h. Supernatant were then collected and analysed by LEGENDplex. Cytokine production (pg/µL) was quantified in unstimulated condition. All results are expressed as mean ± SEM and compared using unpaired t-test.

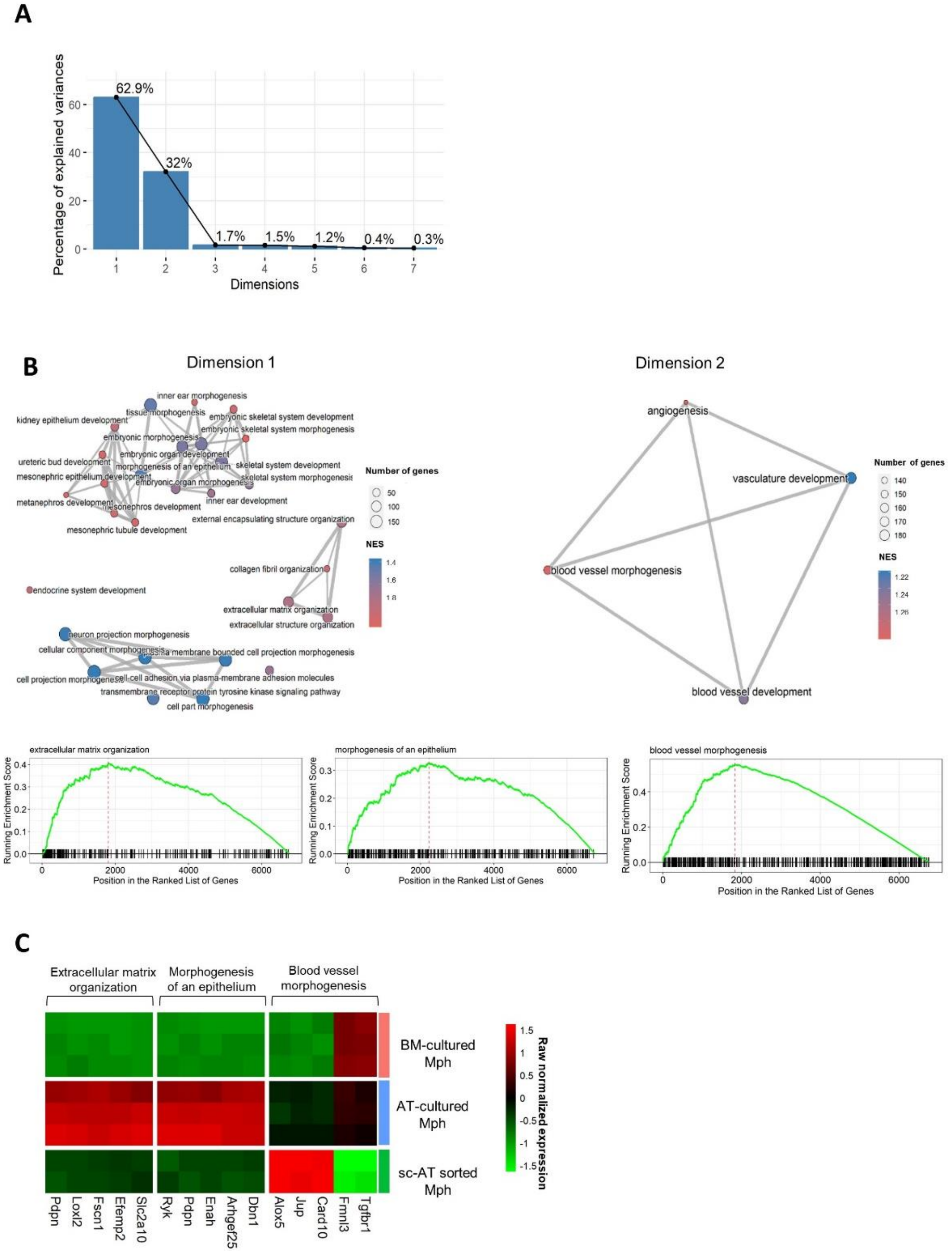

Supplementary Figure 5: Comparison of AT- and BM- cultured macrophages with sc-AT macrophages by bulk RNAseq analysis

Analysis of bulk RNAseq performed on mouse sorted sc-AT macrophages, AT- and BM- cultured macrophages **A**. Scree plot showing the variance explained by each component in PCA, between the three conditions. **B.** Gene set enrichment analysis (GSEA) performed for each dimension, utilizing the PCA contribution as a ranking function for GO BP exploration (upper panel). Enrichment plots for GO processes : “extracellular matrix organization”, “morphogenesis of an epithelium”, and “blood vessel morphogenesis” (lower panel). **C.** Heatmap of the top 5 contributing genes for the 3 GO processes identified in GSEA analysis.

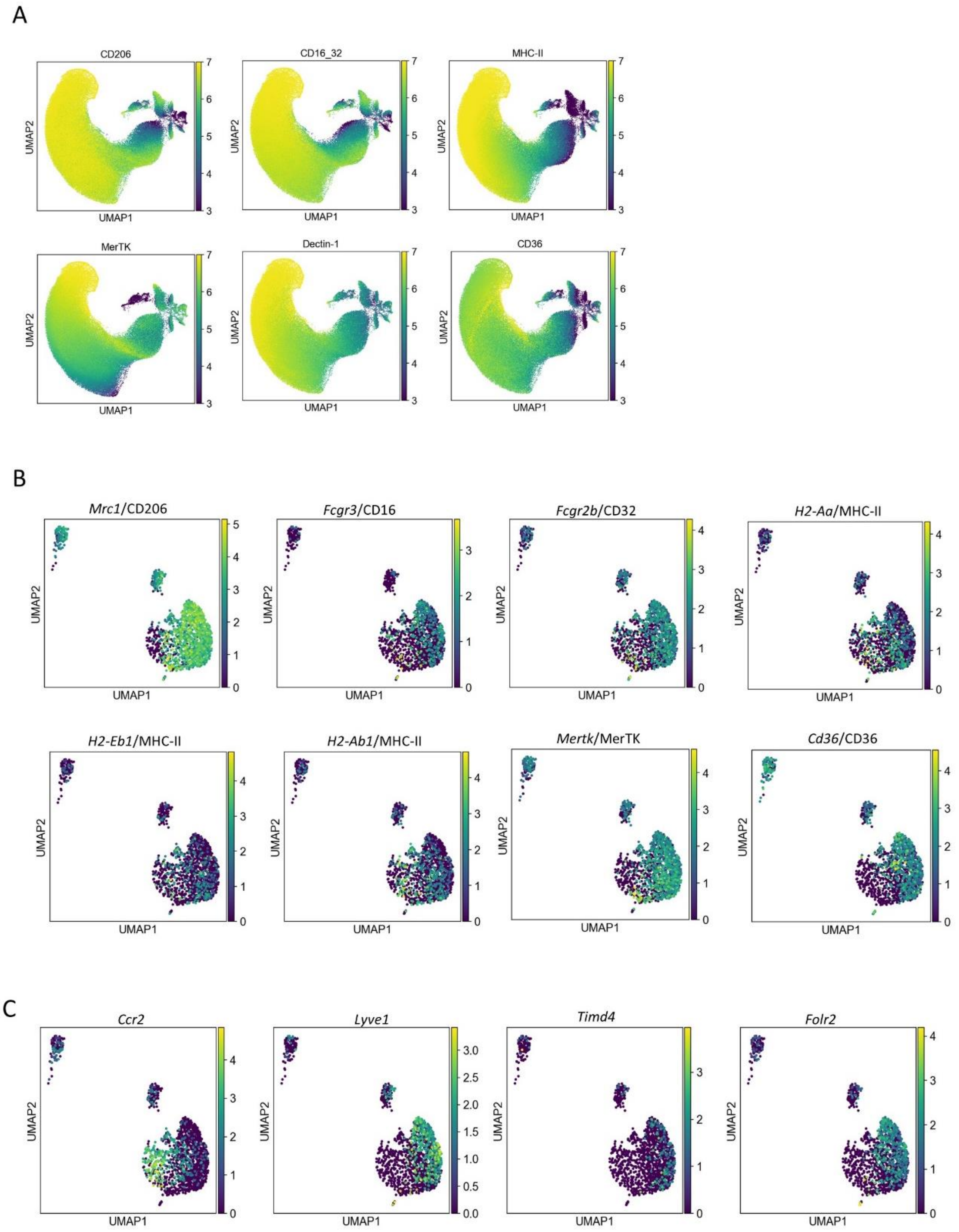

Supplementary Figure 6: UMAP plots of in vivo sc-AT macrophages

A. Unbiased analysis of SVF cells obtained from mouse sc-AT was realised by flow cytometry. The protein expression patterns projected onto a UMAP plots of CD206, CD16/32, Dectin-1, MHC-II, MerTK and CD36 (scale: log-transformed gene expression).**B and C**. Single cell RNAseq analysis of sc-AT macrophages extracted from Emont *et al.* dataset (#GSE176171) (GSM5820690_Mm_ING_08-3). **B.** Gene expression patterns projected onto a UMAP plots of *Mrc1*, *Fcgr3*, *Fcgr2b*, *Mertk*, *H2-Aa*, *H2-Eb1*, *H2-Ab1*, *Cd36* (scale : log-transformed gene expression) **C**. Gene expression patterns projected onto a UMAP plots of of *Ccr2*, *Lyve1*, *Timd4* and *Folr2* (scale : log-transformed gene expression).

1.2 Supplementary Tables

Supplementary Table 1: Antibodies for cytometry staining

Antibody	Fluorescence	Provider	Clone
CCR2	PE Vio-770	Miltenyi Biotec	REA538
CD11b	PerCPCy5.5	BD Pharmigen	M1/70
CD11b	BUV737	BD Horizon	M1/70
CD11c	PE	Miltenyi Biotec	REA754
CD16/32	APC-Cy7	BD Pharmigen	2.4G2
CD16/32	FITC	BD Pharmigen	2.4G2
CD206	APC	eBioscience	MR6F2
CD206	BV605	Biolegends	C068C2
CD24	APC	BD Pharmigen	M1/69
CD29	PE Vio-770	Miltenyi Biotec	HMb1-1
CD31	PE	BD Pharmigen	MEC13-3
CD36	PE	Biolegends	HM36
CD45	APC-Cy7	BD Pharmigen	30F11
CD45	BV786	BD Horizon	30F11
CD90	FITC	BD Pharmigen	53-2.1
Dectine-1	FITC	Miltenyi Biotec	REA263
F4/80	PECy7	eBioscience	BM8
F4/80	BV711	BD Horizon	T45-2342
Ly6C	FITC	BD Pharmigen	AL-21
Ly6G	APC	BD Pharmigen	1A8
Lyve-1	eFluor 660	eBiosciences	ALY7
MerTK	BV650	BD OptiBuild	108928
MHC-II	V500	BD Horizon	M5-114-15.2
MHC-II	Vioblue	Miltenyi Biotec	M5-114-15.2
TIM-4	PE	Miltenyi Biotec	REA999
Lin	PerCP-Cy5.5	BD Pharmigen	145-2C11, M1/70, RA3-6B2, TER-119, RB6-8C5
Ly-6A/E (Sca-1)	V500	BD Horizon	D7
c-Kit (CD117)	PE-Cy7	BD Pharmigen	2B8
CD34	AlexaFluor 647	BD Pharmigen	RAM34

Supplementary Table 2: Murine primer sequences used in RT-qPCR analysis

Gene	Forward primer	Reverse primer
Arginase-1	CGT-GTA-CAT-TGG-CTT-GCG-AG	TCG-GCC-TTT-TCT-TCC-TTC-CC
iNOS	TCC-TGG-ACA-TTA-CGA-CCC-CT	ACA-AGG-CCT-CCA-ATC-TCT-GC
